# Coherent periodic activity in excitatory neural networks : the role of network connectivity

**DOI:** 10.1186/1471-2202-12-S1-P242

**Published:** 2011-07-18

**Authors:** Alessandro Torcini, Lorenzo Tattini, Simona Olmi

**Affiliations:** 1 CNR – Consiglio Nazionale delel Ricerche – Istituto dei Sistemi Complessi, 50019 Sesto Fiorentino, Italy; 2 INFN Sez. Firenze, via Sansone,1 – 50019 Sesto Fiorentino, Italy

## 

Neural collective oscillations have been observed in very many context in brain circuits, ranging from ubiquitous

*γ*-oscillations to *θ*-rythm in the hyppocampus. The origin of these oscillations is commonly associated to the balance between excitation and inhibition in the network, while purely excitatory circuits are believed to lead to “unstructured population bursts” [1]. However, coherent activity patterns have been observed also in “in vivo” measurements of the developing rodent neocortex and hyppocampus for a short period after birth, despite the fact that at this early stage the nature of the involved synapses is essentially excitatory [2]. Of particular interest are the so-called giant depolarizing potentials (GDPs), recurrent oscillations which repetitevely synchronizes a relatively small assembly of neurons and whose degree of synchrony is orchestrate by hub neurons [3]. Furthermore, in their recent study Bonifazi et al. [3] found that the functional connectivity of developing hippocampal networks is characterized by a power-law distribution of the output links with an exponent *δ* ~ 1.1 – 1.3.

On the other hand, computational and theoretical studies of excitatory networks of leaky integrate-and-fire (LIF) neurons have revealed a regime characterized by coherent periodic activity at a macroscopic level both in fully [4] as well as in diluted networks [5]. This regime is characterized by a *partial synchronization* (PS) among the neurons reflected by a periodic behaviour of collective observables, while the single neuron evolution is quasi-periodic in time. The random dilution in the neuronal connectivity induces a weak form of chaos which vanishes for sufficiently large networks, therefore the PS regime appears to be robust with respect to this kind of disorder [6].

In the same framework, namely pulse-coupled LIF excitatory networks, we have analyzed the role of the average degree of connectivity for the stability of the PS regime. In particular, we have considered directed random network with Erdös-Renyi topology with an average connectivity C corresponding to that of a *finite scale-free network* characterized by a power-law distribution with a decay exponent 1 <*δ* < 2. This amounts to have C which scales as N^(2-*δ*) with the number of neurons in the network. Therefore as two limiting cases massively connected networks are recovered for *δ* = 1 (where C is proportional to N) and for *δ*=2 sparse networks (where C is constant). Our analysis has revealed that, for a fixed system size N, coherent periodic dynamics can be observed only for a connectivity C larger than a critical value Г=Г(N) and that this value is independent from the exponent *δ*. Furthermore, in the thermodynamic limit Г tends to an asymptotic value which depends only how the random connections are generated, namely statically or dynamically. The emergence of collective dynamics is controlled simply by the number of afferent synapses, independent of all the other details in the network topology, in agreement with what found for the synchronization of bursting neurons [6].

Finally, we have found indications that these systems are weakly chaotic, in the sense that in the thermodynamic limit completely regular dynamical behaviours are recovered [5]. Nonetheless, the degree of chaoticity increases by increasing *δ* and in the sparse network limit we expect to observe a constant maximal Lyapunov exponent, independent by N.
